# Orbital Doppler Ultrasonography and Optic Nerve Sheath Diameter in Pediatric Brain Death Evaluation

**DOI:** 10.3390/jcm15083156

**Published:** 2026-04-21

**Authors:** Mehmet Ali Durmuş, Alper Karacan, Onur Taydaş, Mehmet Özgür Arslanoğlu, Zeynep Yıldız, Onur Paşa, Sinan Taşdoğan, Tunahan Dertli, Laçin Tatlı Ayhan, Mustafa Özdemir, Mehmet Halil Öztürk

**Affiliations:** 1 Department of Radiology, Sakarya University Training and Research Hospital, 54100 Sakarya, Turkey; alperkaracan@sakarya.edu.tr (A.K.); taydasonur@gmail.com (O.T.); sinantasdogan@gmail.com (S.T.); tunahandertli97@gmail.com (T.D.); drlacintatli@gmail.com (L.T.A.); drmstfrd@gmail.com (M.Ö.); ozturkmh@gmail.com (M.H.Ö.); 2 Department of Pediatric Intensive Care, Sakarya University Training and Research Hospital, 54100 Sakarya, Turkey; ozgurarslanoglu@yahoo.com.tr; 3 Department of Radiology, Gaziantep City Hospital, 27470 Gaziantep, Turkey; yildizzeynep839@gmail.com; 4 Department of Radiology, Ağrı Training and Research Hospital, 04100 Ağrı, Turkey; onur_pasa84@hotmail.com

**Keywords:** brain death, pediatric, orbital Doppler ultrasonography, optic nerve sheath diameter, resistive index, diagnostic accuracy, point-of-care ultrasound, intracranial pressure

## Abstract

**Background/Objectives**: Brain death determination in children is clinically challenging. When standard clinical examination cannot be completed or reliably interpreted, ancillary testing is required—yet many established methods depend on infrastructure or patient transport that may not be feasible in critically ill pediatric patients. Orbital ultrasonography is bedside-applicable and non-invasive, but remains poorly characterized in children. **Methods**: We conducted a single-center retrospective study of 28 pediatric patients evaluated for suspected brain death between January 2021 and February 2025. Patients were classified as brain death-positive [BD(+), *n* = 20] or brain death-negative [BD(−), *n* = 8] based on clinical criteria independent of imaging findings. Orbital color Doppler parameters (ophthalmic artery, central retinal artery, posterior ciliary artery) and optic nerve sheath diameter (ONSD) were measured under a standardized protocol by a single experienced operator. Ophthalmic artery resistive index (OA-RI) was defined a priori as the primary outcome; ONSD was the secondary outcome. Group comparisons used the Mann–Whitney U test with Cliff’s delta effect sizes; false discovery rate correction was applied to secondary and exploratory comparisons. ROC analyses were performed to assess discriminative performance. The study was reported in accordance with the STARD 2015 guidelines for diagnostic accuracy research. **Results**: OA-RI was markedly higher in BD(+) patients (0.84 [IQR 0.80–0.90] vs. 0.65 [0.58–0.69]; *p* < 0.001; δ = 0.975). ROC analysis yielded an AUC of 0.99 (95% CI: 0.96–1.00); at a cut-off of ≥0.77, sensitivity was 95.0% and specificity 100.0%. ONSD also differed significantly between groups (4.75 [4.15–5.08] mm vs. 3.90 [3.40–4.15] mm; *p* = 0.012; δ = 0.619; AUC = 0.81, 95% CI: 0.62–1.00; cut-off ≥ 4.2 mm; sensitivity and specificity both 75.0%). Across all three orbital vessels, end-diastolic velocity was consistently reduced and resistive indices elevated in BD(+) patients. Systolic velocities did not differ meaningfully between groups. Cut-off values represent cohort-specific statistical optima and should be interpreted as exploratory. **Conclusions**: Orbital Doppler ultrasonography demonstrates a coherent high-resistance hemodynamic pattern in pediatric brain death. OA-RI showed strong discriminative performance and may serve as a useful bedside adjunct in selected cases where ancillary testing is indicated. ONSD provides complementary anatomical evidence. These findings are exploratory and require prospective validation in larger, multicenter pediatric cohorts.

## 1. Introduction

Brain death represents the irreversible cessation of all brain and brainstem function and is recognized as the definitive medical determination of death. Accurate, timely diagnosis carries implications that extend well beyond intensive care management—it shapes ethical decision-making, communication with families, and the logistics of organ donation. In pediatric patients, the process is more demanding still: age-related physiological variation, the inherent difficulty of interpreting neurological examination findings in children, and the need to rigorously exclude reversible conditions all add layers of complexity to clinical assessment [1,2,3].

The diagnosis rests primarily on clinical examination. Deep coma, loss of brainstem reflexes, and a positive apnea test together form the diagnostic foundation. Yet full consensus across guidelines remains elusive—particularly in pediatric practice, where observation periods, the need for repeat examinations, and the role of ancillary testing are defined inconsistently across institutions. The World Brain Death Project proposed a broader international framework for brain death/death by neurologic criteria, but heterogeneity in the indications for and selection of ancillary tests persists in practice—largely because high-quality pediatric validation studies remain scarce, leaving clinicians without robust evidence to guide test selection in children [4]. The 2023 joint pediatric and adult consensus guideline addressed this gap to some extent, providing more granular guidance on the second examination, observation periods, and ancillary test requirements for specific age groups; however, it underscored that the need for standardization in this field remains far from resolved [2].

When clinical evaluation cannot be completed or reliably interpreted, ancillary testing becomes necessary. Electroencephalography, transcranial Doppler ultrasonography, radionuclide scintigraphy, digital subtraction angiography, CT angiography, and MR-based perfusion or flow studies represent the principal modalities employed for this purpose [2,4,5]. Transcranial Doppler ultrasonography is the most closely related bedside modality; however, its utility may be limited by inadequate temporal acoustic windows, and it requires specialized probes not universally available. Many of these tests, however, demand specialized infrastructure, require patient transport, or prove impractical in hemodynamically unstable children. Compounding this, the apnea test cannot always be safely completed in pediatric patients, the effects of pharmacological agents may be unpredictable, and the risks of transporting critically ill children are non-trivial—all of which further increase the need for bedside-available supportive methods [1,2,3].

Orbital ultrasonography offers a practical alternative in this context. It is usable at the bedside, non-invasive, and can be repeated within minutes. The technique provides two complementary categories of information: hemodynamic parameters, derived from spectral Doppler interrogation of the orbital arteries, which reflect the peripheral consequences of cerebral circulatory compromise; and structural markers, specifically optic nerve sheath diameter (ONSD), which reflect the morphological impact of raised intracranial pressure. Prior studies suggest that the method may carry adjunctive diagnostic value in the setting of brain death and intracranial hypertension [6,7,8,9,10,11].

The existing literature, however, is both limited and heterogeneous. Some studies have focused on a single orbital vessel; others have examined optic nerve sheath diameter (ONSD) in isolation. Most available evidence derives from adult cohorts with small sample sizes, and pediatric-specific validation studies remain sparse—limiting the direct applicability of existing findings to children [8,9,12]. Age-related variability in normative ONSD distribution adds another layer of difficulty, complicating the interpretation of threshold values in children [7,10,13]. Against this background, the present study evaluated orbital color Doppler ultrasonography parameters—ophthalmic artery (OA), central retinal artery (CRA), and posterior ciliary artery (PCA)—alongside optic nerve sheath diameter, in pediatric patients under evaluation for suspected brain death. The primary outcome was the discriminative performance of the ophthalmic artery resistive index (OA-RI), whereas the complementary value of ONSD was examined as the secondary outcome. We hypothesized that OA-RI would be significantly elevated in patients with brain death compared to those without, reflecting the increased downstream vascular resistance that accompanies cerebral circulatory arrest. The intended clinical role of orbital Doppler in this context is supportive rather than definitive: to provide confirmatory bedside evidence consistent with cerebral circulatory arrest in cases where standard ancillary testing faces practical limitations, rather than to independently establish or exclude brain death.

## 2. Materials and Methods

This was a single-center, retrospective, observational study. Patients under 18 years of age who underwent orbital color Doppler ultrasonography (US) for suspected brain death between 1 January 2021 and 1 February 2025 were identified through a retrospective review of institutional records. The study protocol was approved by the Sakarya University Health Sciences Scientific Research Ethics Committee on 21 February 2025 (decision no. E/43012747-050.04-459105-159). Given the retrospective design, the requirement for informed consent was waived. The study was conducted in accordance with the Declaration of Helsinki and was reported in accordance with STARD 2015 (Table S1).

Institutional archive records were screened to identify patients who had undergone orbital Doppler US in the context of suspected brain death. Inclusion required a technically adequate orbital Doppler examination, availability of recorded spectral Doppler parameters, and presence of an optic nerve sheath diameter measurement. Patients with missing measurement data, insufficient image quality, or overt orbital pathology that could compromise measurement reliability were excluded. No imputation was applied for missing data; only cases with a complete measurement set were included. A total of 28 patients met these criteria and were included in the analysis.

Patients were classified into two groups according to the final clinical assessment documented in the medical records, independently of imaging findings. The brain death-positive group [BD(+), *n* = 20] comprised patients in whom absence of brainstem reflexes and a positive apnea test had been documented by the responsible clinical physicians. All BD(+) determinations were made in accordance with Turkish national brain death guidelines (2022 legislation) by a two-physician committee consisting of one neurologist or neurosurgeon and one anesthesiologist or intensivist, as mandated by national regulations. CTA was performed in 16 of 20 BD(+) patients (80%) on the same day or the following day, confirming absence of cerebral arterial circulation; the remaining 4 patients died before imaging could be completed. Under Turkish guidelines, demonstration of absent cerebral blood flow on ancillary testing allows waiver of the otherwise required observation period. No borderline or delayed determinations were present; all BD(+) patients demonstrated unequivocal clinical findings at initial evaluation. The brain death-negative group [BD(−), *n* = 8] consisted of patients who had undergone orbital Doppler US for suspected brain death but did not meet clinical criteria on final evaluation. This group was not composed of healthy controls; rather, these were critically ill patients monitored in the intensive care unit who had been clinically assessed for brain death due to severe neurological injury. Clinical characteristics and admission diagnoses of the study population are summarized in Table 1.

Orbital Doppler US and the clinical reference assessment were performed within the same clinical time window. According to the chart documentation, the apnea test and orbital Doppler examination were performed on the same day and in close temporal proximity.

All ultrasonographic examinations were performed using a Samsung HM70 EVO portable ultrasound system (Samsung Medison Co., Ltd., Seoul, Republic of Korea) with an LA3-16AD linear array transducer (Samsung Medison Co., Ltd., Seoul, Republic of Korea) (center frequency approximately 10 MHz; operating range 3–16 MHz). Patients were examined in the supine position; measurements were obtained through closed eyelids with minimal probe pressure to avoid elevation of intraorbital pressure. The spectral Doppler sample volume was positioned with appropriate angle correction, and technical standardization was maintained throughout. According to archive records, all examinations had been performed by a single operator with approximately five years of experience; accordingly, inter-observer variability analysis was not applicable, and the retrospective nature of the archived data precluded formal reproducibility assessment.

Optic nerve sheath diameter was measured in the axial plane, perpendicular to the optic nerve axis, at 3 mm posterior to the posterior wall of the globe, and recorded in millimeters. A single eye was analyzed per patient, without right-left comparison, in order to preserve the assumption of independent observations. Because routine archived measurements were consistently available for the right eye, right-eye data were used for analysis. Clinically significant lateral asymmetry is not expected in this context: cerebral circulatory arrest affects both internal carotid arteries symmetrically, and the resulting hemodynamic changes in the orbital vessels—as well as ONSD elevation from raised intracranial pressure—are expected to manifest bilaterally.

Color and spectral Doppler assessment was performed at the level of the OA, CRA, and PCA. Peak systolic velocity (PSV), end-diastolic velocity (EDV), and resistive index (RI) were recorded for each vessel. The systolic-to-diastolic ratio (S/D) was calculated for descriptive completeness but was not included in the primary comparative analysis, because it provides hemodynamic information that is largely redundant with RI. The primary focus of hemodynamic evaluation was on changes in the diastolic flow component and resistance parameters. Negative EDV values and consequently supranormal RI values (>1) were not treated as data errors; these findings were interpreted as potential hemodynamic manifestations of severely elevated distal resistance with reversed or absent diastolic flow.

The statistical analysis plan followed a pre-specified hierarchical framework defined before inspection of the data. The primary analytic objective was not to characterize deviation of imaging parameters from normative values reported in the literature, but to evaluate discriminative performance between BD(+) and BD(−) groups as defined by the clinical reference standard. Ophthalmic artery resistive index (OA-RI) was designated the primary outcome. The rationale for this selection was that the ophthalmic artery, as the principal feeding vessel of the orbital circulation, was expected to reflect intracranial hemodynamic changes more stably and with greater technical reliability than distal vessels. Higher measurement reproducibility and easier standardization in clinical practice were additional considerations in designating OA-RI as the primary endpoint.

ONSD was designated as the secondary outcome because it represents a complementary anatomical marker of raised intracranial pressure, operating at a structural rather than hemodynamic level, and was therefore evaluated alongside the Doppler parameters.

The remaining Doppler parameters were treated as exploratory. CRA and PCA measurements were not positioned within the primary hypothesis, as the smaller caliber of these distal vessels renders them more susceptible to technical variability and increased measurement error.

Continuous variables are reported as median and interquartile range (IQR). Given the limited sample size and the inability to reliably satisfy parametric assumptions, the Mann–Whitney U test was used for between-group comparisons. Effect sizes were quantified using Cliff’s delta (δ), a non-parametric measure ranging from −1 to +1. Following standard conventions, |δ| < 0.33 was considered a small effect, 0.33–0.47 medium, and >0.47 large; values approaching ±1 indicate near-complete separation, such that virtually any BD(+) patient would have a higher value than any BD(−) patient, or vice versa. For pre-specified secondary and exploratory imaging parameters, false discovery rate (FDR) correction was applied using the Benjamini–Hochberg method to reduce the impact of multiple comparisons.

ROC curves were constructed and the area under the curve (AUC) was calculated for the primary and secondary parameters. Optimal cut-off values were determined using the Youden J index. Given the small sample size, ROC findings were interpreted as exploratory rather than confirmatory. A two-sided *p*-value of <0.05 was considered statistically significant throughout. Analyses were performed using SPSS version 29 (IBM Corp., Armonk, NY, USA). During the preparation of this manuscript, Claude (Anthropic, San Francisco, CA, USA; claude.ai, accessed March 2025) was used for language editing purposes. The authors have reviewed and edited the output and take full responsibility for the content. No a priori sample size calculation was conducted, consistent with the retrospective, archive-based study design. All eligible patients identified during the study period who met the inclusion criteria and had no exclusion criteria were enrolled consecutively; the sample size was thus determined by the available case pool.

## 3. Results

A total of 28 pediatric patients were included in the analysis: 20 BD(+) and 8 BD(−). Age distributions for both groups are reported as median and interquartile range. Ultrasonographic measurements were obtained from a single eye in all patients, with no right-left distinction applied. Demographic and measurement characteristics are presented in Table 2.

Between-group comparisons followed the pre-specified hierarchical analysis plan. OA-RI, the primary outcome, was significantly higher in BD(+) patients than in BD(−) patients (0.84 [0.80–0.90] vs. 0.65 [0.58–0.69]; *p* < 0.001), with an effect size of δ = 0.975—indicating near-complete separation between groups. As the pre-specified primary endpoint with the strongest discriminative performance in this cohort, OA-RI emerged as the most robust Doppler parameter.

ONSD also differed significantly between groups (4.75 [4.15–5.08] mm vs. 3.90 [3.40–4.15] mm; *p* = 0.012; δ = 0.619), reflecting moderate-to-strong separation. The difference remained significant after FDR correction (q = 0.027), suggesting that ONSD provides additional discriminative information beyond the primary Doppler finding.

Within the exploratory analysis, the most pronounced differences were observed in diastolic flow-related parameters. OA-EDV was significantly lower in BD(+) patients (*p* = 0.001; δ = −0.800; q = 0.003), as was PCA-EDV (*p* < 0.001; δ = −0.838; q = 0.002). CRA-EDV was also reduced in the BD(+) group, though it remained borderline after FDR correction (*p* = 0.027; q = 0.050).

Resistive indices followed the same pattern: CRA-RI (δ = 0.856; q = 0.002) and PCA-RI (δ = 0.788; q < 0.001) were markedly elevated in BD(+) patients. These findings were nonetheless interpreted within a secondary and exploratory framework, given the greater susceptibility of distal vessel measurements to technical variability.

Peak systolic velocity showed little discriminative value across all vessels (OA-PSV *p* = 0.899; CRA-PSV *p* = 0.508; PCA-PSV *p* = 0.067). The overall Doppler hemodynamic pattern in BD(+) patients was thus consistently characterized by reduced diastolic flow and elevated vascular resistance.

In a subset of BD(+) patients, negative EDV values and RI values exceeding 1.0 were recorded. Specifically, negative EDV—indicating reversed diastolic flow—was observed in 3 of 20 BD(+) patients (15%). As noted in the Methods, these were not treated as data errors but as hemodynamic manifestations of severely elevated distal resistance with reversed or absent diastolic flow, consistent with prior descriptions of diastolic flow reversal in brain death [8,14]. Detailed statistical results for all between-group comparisons are presented in Table 3.

**Figure 1:**
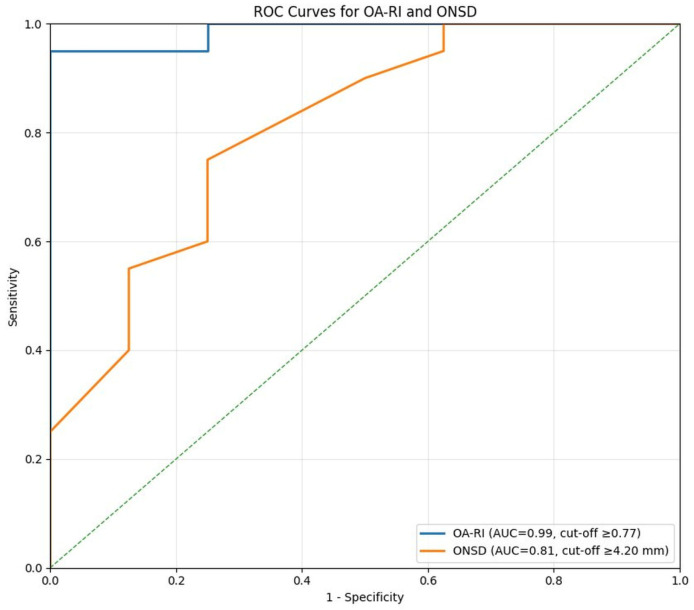
ROC curves for OA-RI and ONSD. OA-RI demonstrated the highest discriminative performance for brain death evaluation (AUC = 0.99; 95% CI: 0.96–1.00; cut-off ≥ 0.77). ONSD showed more limited but balanced discriminative performance (AUC = 0.81; 95% CI: 0.62–1.00; cut-off ≥ 4.2 mm). Cut-off values represent cohort-specific statistical optima and should not be interpreted as generalizable clinical decision thresholds. The diagonal dashed line represents the reference line of no discrimination (AUC = 0.5).

ROC analyses were performed to assess the discriminative performance of the primary and secondary parameters (Figure 1). OA-RI yielded an AUC of 0.99 (95% CI: 0.96–1.00); the Youden J index identified an optimal cut-off of ≥0.77, at which sensitivity was 95.0% and specificity 100.0%. However, the small size of the BD(−) group limits the generalizability of this threshold, which should be interpreted as a cohort-specific statistical optimum rather than a broadly applicable clinical decision boundary. Although the 95% confidence interval appears narrow (0.96–1.00), this apparent precision should be interpreted with caution: in small samples, confidence intervals may underestimate true uncertainty, and the near-perfect AUC likely reflects, at least in part, the limited sample size and potential for overfitting rather than confirmed clinical discriminative ability. For ONSD, AUC was 0.81 (95% CI: 0.62–1.00); the wide confidence interval reflects substantial uncertainty in this estimate, consistent with the moderate effect size (δ = 0.619) and the modest sample size. At a cut-off of ≥4.2 mm, both sensitivity and specificity were 75.0%. Rather than a standalone discriminator, ONSD appears better characterized as a complementary anatomical parameter that adds to the information provided by OA-RI. Full ROC results are summarized in Table 4.

## 4. Discussion

This study evaluated the relationship between orbital color Doppler ultrasonography parameters and the clinical reference standard in pediatric patients under evaluation for suspected brain death. The findings reveal a consistent hemodynamic pattern in BD(+) patients, characterized by a marked reduction in diastolic flow and a corresponding rise in resistance parameters. Within this pattern, OA-RI—the pre-specified primary outcome—proved the most robust parameter both in terms of statistical separation and effect size. ONSD contributed complementary anatomical evidence, supporting the hemodynamic findings at a structural level. Taken together, these findings suggest that the high-resistance pattern identified on orbital Doppler examination is pathophysiologically consistent with brain death [6,8,9,11].

The pathophysiology of cerebral circulatory arrest in brain death follows a well-characterized sequence. As intracranial pressure (ICP) rises and approaches mean arterial pressure, cerebral perfusion pressure (CPP = MAP − ICP) progressively declines. When ICP equals or exceeds MAP, forward cerebral blood flow ceases. This process does not occur instantaneously but evolves through recognizable hemodynamic stages that can be detected on Doppler examination.

On transcranial Doppler (TCD), this progression manifests as: (1) increased pulsatility with preserved flow, (2) sharp systolic spikes with markedly reduced or absent diastolic flow, (3) oscillating (to-and-fro) flow representing systolic forward and diastolic retrograde movement, and ultimately (4) absent flow signal. These TCD patterns are well established as markers of cerebral circulatory arrest and form the basis for TCD’s role as an ancillary test in brain death determination.

**Figure 2:**
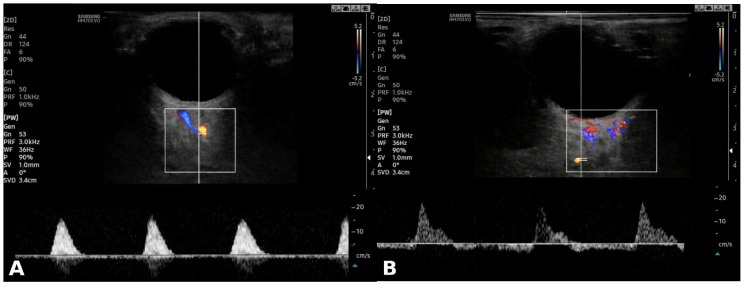
Representative orbital spectral Doppler waveforms illustrating the high-resistance pattern associated with brain death. (**A**) Short systolic spike with marked loss of diastolic flow. (**B**) Alternating/reversed flow pattern. The colored boxes indicate the spectral Doppler sample volume placement at the level of the ophthalmic artery.

Orbital Doppler examination interrogates this same hemodynamic process from a peripheral vantage point. The ophthalmic artery originates directly from the internal carotid artery and thus reflects intracranial hemodynamic conditions. As cerebral vascular resistance rises to supraphysiological levels, the orbital vessels—which share a common arterial inflow—demonstrate parallel changes: progressive diastolic flow reduction, elevated resistive indices, and in advanced cases, oscillating or reversed diastolic flow [8,9,14,15,16]. The EDV reduction observed in BD(+) patients—progressing to negative values in some cases—and the corresponding rise in RI, including supranormal values exceeding 1.0 in a subset, are consistent with this pathophysiological model. Representative spectral Doppler waveforms illustrating the high-resistance pattern are shown in Figure 2.

The concordance between TCD and orbital Doppler patterns is not coincidental but reflects their shared anatomical and physiological substrate. However, orbital Doppler offers practical advantages: it does not require an acoustic window through the temporal bone (which may be inadequate in a subset of patients), and it can be performed with standard portable ultrasound equipment without specialized TCD probes.

The stronger discriminative performance of OA-RI relative to other orbital Doppler parameters likely reflects two complementary advantages: the ophthalmic artery is the principal feeding vessel of the orbital circulation, and it can be sampled with greater technical reliability. Spectral waveforms in the OA tend to be more stable, whereas the smaller caliber of distal vessels such as the CRA and PCA introduces measurement variability that can obscure diastolic parameters in particular. That OA-RI emerged as the strongest Doppler marker in this cohort is therefore consistent with both the anatomical and technical characteristics of these vessels, and with the existing literature [6,8,15,17].

Our findings are broadly consistent with the existing literature, both in terms of pattern and quantitative direction. Karaali et al. described diastolic flow loss or reversal at the level of the OA and central retinal artery in adult brain death cases, with RI values approaching 1.0, as a characteristic hemodynamic signature [8]. Algin et al. compared orbital Doppler findings with transcranial Doppler and reported that RI ≥ 1 on CRA-based assessment supported the high-resistance pattern, while cases with RI < 1 warranted additional confirmation [6]. In a more recent prospective, single-blind study, Ökmen et al. demonstrated strong agreement between OA-based orbital Doppler measurements and CT angiography, with high overall diagnostic performance [15]. On the pediatric side, Riggs et al. highlighted spectral changes in distal orbital vessel patterns—specifically diastolic flow loss, short systolic spikes, and oscillatory waveforms [9]. Aslan et al. showed that combining orbital Doppler findings with ONSD measurement may strengthen diagnostic discrimination in pediatric patients [12]. The same pathophysiological direction was evident in our cohort—yet the strongest and most clinically tractable discrimination was obtained at the level of the proximal vessel, the ophthalmic artery, and specifically through OA-RI. This finding advances the existing literature in several respects. First, it systematically evaluates multiple orbital vessels alongside ONSD within a single pediatric cohort under standardized conditions. Second, it identifies OA-RI—rather than distal vessel parameters—as the most robust discriminator, which has practical implications for clinical implementation. Third, it employs a pre-specified hierarchical analytical framework with effect size quantification and multiple comparison correction, providing more rigorous methodology than most prior studies in this field.

PCA and CRA findings corroborated the high-resistance pattern. Reduced diastolic flow and elevated RI in these vessels suggest that the peripheral orbital circulation is affected in a similar fashion. Their smaller caliber and greater technical difficulty in sampling, however, introduce measurement variability that must be taken into account [17]. Distal vessel findings should therefore be regarded not as primary markers, but as exploratory indicators that lend support to OA-RI [8,9].

ONSD reflects the structural consequences of raised intracranial pressure rather than flow dynamics directly and should therefore be interpreted as a complementary marker rather than an alternative to OA-RI. The significant between-group difference observed in our cohort is consistent with prior pediatric studies evaluating ONSD in critically ill children [10,11,12,18]. The ROC-derived cut-off of ≥4.2 mm also aligns more closely with pediatric than adult thresholds reported in the literature. In children, Bansal et al. reported a cut-off of approximately 4.46 mm with good discriminative performance [19], whereas Çevikkalp et al. reported a substantially higher threshold in an adult cohort [20]. This difference likely reflects the distinct normative ONSD distributions seen across pediatric and adult populations, as also supported by age-dependent pediatric reference data reported by Rizqiamuti et al. [10]. Importantly, ONSD varies not only between pediatric and adult populations but also across pediatric age groups. Normative ONSD values increase with age during childhood, and thresholds derived from mixed-age pediatric cohorts may not be equally applicable to infants, young children, and adolescents. Our cohort spanned a wide age range (median 5.0 years in BD(+), IQR 1.0–9.0), but the limited sample size precluded meaningful age-stratified analysis—stratification into subgroups (e.g., <2 years, 2–6 years, >6 years) would have yielded subsets too small for reliable statistical comparison. Accordingly, the ≥4.2 mm threshold identified in our cohort should be interpreted in an age- and context-sensitive manner rather than as a universal reference point. Combining OA-RI and ONSD in a multimodal diagnostic approach may theoretically enhance discriminative performance by integrating hemodynamic and structural evidence; however, formal multivariate modeling was not pursued in the present study, as the small cohort size would preclude reliable parameter estimation. Future studies with larger sample sizes should explore whether combined algorithms offer incremental diagnostic value over single-parameter assessment, and incorporate age-stratified analyses to establish age-appropriate ONSD thresholds for pediatric brain death evaluation. Selected comparisons with previous studies are summarized in Table 5.

The study has several methodological strengths. The analysis followed a pre-specified hierarchical framework from the outset—OA-RI as the primary outcome, ONSD as secondary, and remaining Doppler parameters as exploratory. Reporting effect sizes alongside *p*-values, as well as applying FDR correction to secondary and exploratory comparisons, allowed findings to be interpreted in a more controlled and transparent manner. A further strength is the simultaneous assessment of OA, CRA, PCA, and ONSD within the same pediatric cohort under a single standardized protocol, rather than focusing on any one vessel or morphological parameter in isolation.

Although the ROC analysis for OA-RI indicated strong discriminative performance, direct generalization of the derived cut-off is not appropriate given the small BD(−) group. The composition of the comparison group—critically ill patients evaluated for suspected brain death rather than healthy children—may also have contributed to a threshold that does not map directly onto general reference ranges. The ≥0.77 cut-off is relatively close to published normative RI values, which further limits its interpretation as a standalone absolute decision threshold. This study was designed to assess discriminative performance between clinically defined critical patient groups, not to establish normative reference intervals; the ≥0.77 value should accordingly be treated as a cohort-specific exploratory partition point rather than a universal clinical threshold. The same applies to the ONSD cut-off of ≥4.2 mm—it represents a statistical optimum within this pediatric critical care cohort and should not be read as an age- and context-independent absolute threshold.

From a clinical standpoint, it is important to clarify where orbital Doppler examination might realistically fit within current pediatric brain death evaluation algorithms. We do not propose orbital Doppler as a primary screening tool; clinical examination—specifically, the demonstration of absent brainstem reflexes and a positive apnea test—remains the established gold standard and should not be supplanted. Rather, orbital Doppler may serve as a confirmatory bedside adjunct in specific clinical scenarios where standard ancillary testing faces practical limitations. Such scenarios include: patients in whom the apnea test cannot be safely completed due to severe hypoxemia or hemodynamic instability; cases where transcranial Doppler is technically inadequate because of insufficient temporal acoustic windows (a recognized limitation in pediatric patients); and critically ill children for whom transport to radiology suites for CT or MR angiography poses unacceptable risk. In these contexts, a bedside-available, repeatable, non-invasive method that demonstrates hemodynamic findings consistent with cerebral circulatory arrest may provide valuable supportive information. Additionally, serial orbital Doppler assessment could theoretically serve a monitoring role—tracking hemodynamic progression in patients at risk for brain death—although this application was not evaluated in the present study and would require dedicated prospective investigation. It must be emphasized that our findings remain exploratory; the proposed cut-off values require external validation, and incorporation into clinical protocols should await confirmation in larger, multicenter pediatric cohorts.

This study carries several limitations. Sample size is restricted—the BD(−) group in particular is small (*n* = 8), which reduces the precision of diagnostic performance estimates and likely contributes to the near-perfect AUC observed for OA-RI. In diagnostic accuracy studies of this size, even a small number of misclassifications would substantially alter performance metrics. Accordingly, these results should be interpreted as hypothesis-generating rather than indicative of near-clinical applicability; the strong discriminative signal observed requires confirmation in adequately powered prospective studies before any clinical implementation can be considered. From a statistical standpoint, formal resampling techniques such as bootstrapping could provide more robust estimates of performance variability; however, given the small BD(−) sample, such analyses would themselves carry substantial uncertainty. Future studies with adequate sample sizes should employ these methods to more rigorously characterize diagnostic performance. The study is single-center and retrospective. Additionally, brain death determination followed Turkish national guidelines (2022), which differ in minor procedural aspects from the 2023 AAN/AAP/CNS/SCCM consensus—for example, Turkish regulations require a two-physician committee and define age-stratified observation periods that may be waived when ancillary testing confirms absent cerebral blood flow. While the core diagnostic criteria (absent brainstem reflexes, positive apnea test) are aligned with international standards, these procedural differences should be considered when interpreting external applicability. Ultrasonographic measurements are inherently operator-dependent, and no inter-observer or intra-observer variability analysis was performed. Archived examinations were conducted as part of routine clinical care without standardized image storage protocols that would permit reliable blinded re-measurement; accordingly, formal reproducibility assessment was not feasible within this retrospective framework. This represents a notable limitation for an operator-dependent technique, and future prospective studies should incorporate structured reproducibility analyses, including inter-observer agreement, to establish measurement reliability across different operators and clinical settings. Single-time-point measurements preclude assessment of the temporal evolution of hemodynamic changes. The use of critically ill patients rather than healthy children as the comparison group is clinically meaningful, but it may have contributed to cut-off values that do not align directly with general reference ranges. Additionally, potential spectrum bias should be acknowledged: the BD(−) group consisted of critically ill patients who clearly did not meet brain death criteria, rather than borderline or diagnostically ambiguous cases. This composition may have inflated discriminative performance by excluding the more challenging intermediate phenotypes that would be encountered in clinical practice. Finally, given that ONSD varies with age and normative distributions are not fixed in the pediatric population, caution is warranted in generalizing a single cut-off value across all age groups [7,10,17]. Our sample size did not permit formal age-stratified subgroup analysis; accordingly, the ≥4.2 mm threshold should be considered a cohort-level exploratory finding rather than an age-specific recommendation.

## 5. Conclusions

The high-resistance pattern characterized by reduced diastolic flow and elevated RI on orbital Doppler examination appears pathophysiologically consistent with pediatric brain death. Within this pattern, OA-RI stands out as a strong and clinically tractable parameter, while ONSD provides supporting anatomical evidence. CRA and PCA findings add corroborating information, reflecting comparable hemodynamic involvement in the peripheral orbital circulation. Given its bedside applicability, non-invasive nature, and repeatability, orbital ultrasonography may serve as a useful adjunct in selected pediatric cases where ancillary testing is indicated. The findings, and the proposed cut-off values in particular, require prospective validation in larger, multicenter pediatric cohorts. Key areas for future investigation may include development of standardized acquisition protocols to ensure measurement consistency across centers, age-stratified threshold analyses to establish developmentally appropriate cut-off values, inter-operator reliability studies to assess reproducibility across different sonographers and clinical settings, and exploration of multimodal diagnostic approaches combining hemodynamic (OA-RI) and structural (ONSD) parameters.

## Figures and Tables

**Table 1:** Demographic and clinical characteristics of the study population.

Variable	BD(+) (*n* = 20)	BD(−) (*n* = 8)
Age, years, median (IQR)	5.0 (1.0–9.0)	6.0 (2.8–9.0)
Sex, male/female, *n*	11/9	5/3
Primary clinical diagnosis/reason for admission		
Traumatic brain injury, *n* (%)	4 (20.0)	4 (50.0)
Hypoxic–ischemic encephalopathy, *n* (%)	9 (45.0)	0 (0.0)
Intracranial hemorrhage, *n* (%)	3 (15.0)	0 (0.0)
Infectious/inflammatory etiology, *n* (%)	3 (15.0)	1 (12.5)
Other, *n* (%)	1 (5.0)	3 (37.5)
Apnea test positivity, *n* (%)	20 (100)	0 (0)
Loss of brainstem reflexes, *n* (%)	20 (100)	0 (0)

**Table 2:** Demographic and ultrasonographic characteristics of the BD(+) and BD(−) groups.

Parameter	BD(+) (*n* = 20) Median (IQR)	BD(−) (*n* = 8) Median (IQR)
Age (years)	5.00 (0.96–9.00)	6.00 (2.75–9.00)
ONSD (mm)	4.75 (4.15–5.08)	3.90 (3.40–4.15)
Ophthalmic Artery (OA)		
PSV (cm/s)	13.71 (10.07–15.63)	10.95 (10.44–19.12)
EDV (cm/s)	1.85 (1.10–2.38)	4.52 (3.10–6.71)
RI	0.84 (0.80–0.90)	0.65 (0.58–0.69)
S/D	5.35 (4.41–7.70)	2.79 (2.50–3.04)
Central Retinal Artery (CRA)		
PSV (cm/s)	9.38 (5.35–14.21)	12.66 (8.37–22.66)
EDV (cm/s)	1.70 (0.99–2.60)	5.55 (2.54–9.76)
RI	0.80 (0.73–0.88)	0.63 (0.55–0.69)
S/D	4.02 (3.21–5.13)	2.47 (2.03–2.75)
Posterior Ciliary Artery (PCA)		
PSV (cm/s)	10.95 (7.14–14.52)	17.52 (10.20–23.34)
EDV (cm/s)	2.07 (0.95–3.22)	9.96 (3.70–13.97)
RI	0.82 (0.79–0.86)	0.54 (0.46–0.62)
S/D	5.13 (3.93–6.28)	1.92 (1.81–2.36)

**Table 3:** Comparative analysis results between BD(+) and BD(−) groups.

Parameter	*p*-Value	Cliff’s δ	q (FDR)
OA-RI	<0.001	0.975	NA
ONSD	0.012	0.619	0.027
OA-EDV	0.001	−0.800	0.003
CRA-RI	<0.001	0.856	0.002
PCA-RI	<0.001	0.788	<0.001
PCA-EDV	<0.001	−0.838	0.002
CRA-EDV	0.027	−0.550	0.050
PCA-PSV	0.067	−0.456	0.109
OA-PSV	0.899	−0.038	1.000
CRA-PSV	0.508	−0.169	0.734

**Table 4:** ROC analysis results for primary and secondary parameters.

Parameter	AUC	95% CI	Optimal Cut-Off	Sensitivity	Specificity
OA-RI	0.99	0.96–1.00	≥0.77	95.0%	100.0%
ONSD	0.81	0.62–1.00	≥4.2 mm	75.0%	75.0%

**Table 5:** Comparison of present findings with selected literature.

Study	Population/Parameter	Main Finding	Relation to Present Study
Karaali et al. [8]	Adult/OA, CRA Doppler	Diastolic flow loss or reversal; RI approaching 1.0	High-resistance pattern confirmed; strongest discrimination obtained with OA-RI
Algin et al. [6]	Adult/orbital Doppler	RI elevation and high-resistance pattern	Consistent with OA-RI and distal RI findings
Ökmen et al. [15]	Adult/OA Doppler, CTA correlation	Systolic spike + diastolic flow loss pattern; sensitivity 100%, specificity 93%	Strongly consistent with representative waveforms and high-resistance pattern interpretation
Riggs et al. [9]	Pediatric/distal orbital vessel Doppler	Diastolic flow loss, short systolic spike, oscillatory flow	CRA/PCA findings support this pattern; strongest discrimination with OA-RI
Aslan et al. [12]	Pediatric/ONSD + orbital Doppler	Combined assessment may enhance diagnostic discrimination	Supports complementary role of ONSD alongside OA-RI
Bansal et al. [19]	Pediatric/ONSD	Cut-off ~4.46 mm; high discriminative performance	Consistent with ≥4.2 mm cut-off in present cohort
Çevikkalp et al. [20]	Adult/ONSD	Threshold >6.62 mm	Lower threshold in present study consistent with pediatric–adult normative difference
Cour-Andlauer et al. [7]	Pediatric/ONSD	Median 5.58 mm; limited discriminative performance in some clinical contexts	Supports age- and context-sensitive interpretation of ONSD thresholds
Rizqiamuti et al. [10]	Healthy pediatric controls/ONSD	Age-dependent normative ONSD values	Supports context-sensitive interpretation of ≥4.2 mm cut-off

## Data Availability

The data presented in this study are available upon request from the corresponding author due to patient privacy and institutional ethical restrictions.

## Supplementary Materials

The following supporting information can be downloaded at: https://www.mdpi.com/article/10.3390/jcm15083156/s1. Table S1: STARD 2015 Checklist.
