# Supplementary material for: Orbital Doppler Ultrasonography and Optic Nerve Sheath Diameter in Pediatric Brain Death Evaluation
Source: J Clin Med. 2026 Apr 21;15(8):3156. doi: 10.3390/jcm15083156 (PMC13117453; doi:10.3390/jcm15083156)
Supplement: Supplementary file 1 [file jcm-15-03156-s001.zip › jcm-4242569-supplementary.pdf]

STARD 2015 Checklist

Standards for Reporting of Diagnostic Accuracy Studies

Orbital Doppler Ultrasonography and Optic Nerve Sheath Diameter in Pediatric Brain Death Evaluation

Journal of Clinical Medicine — Supplementary Material

No	STARD 2015 Item	Location in Manuscript
**TITLE / ABSTRACT**		
1	Identification as a study of diagnostic accuracy using at least one measure of accuracy (such as sensitivity, specificity, predictive values, or AUC)	Title; Abstract (Results): AUC, sensitivity, specificity reported
**ABSTRACT**		
2	Structured summary of study design, methods, results, and conclusions	Abstract: Background / Methods / Results / Conclusions
**INTRODUCTION**		
3	Scientific and clinical background, including the intended use and clinical role of the index test	Introduction, paragraphs 1–5: brain death determination challenges, limitations of established ancillary tests, rationale for orbital ultrasonography
4	Study objectives and hypotheses	Introduction, final paragraph: discriminative performance of OA-RI as primary outcome; ONSD as secondary outcome
**METHODS — Study Design**		
5	Whether data collection was planned before the index test and reference standard were performed (prospective study) or after (retrospective study)	Materials and Methods, paragraph 1: 'single-center, retrospective, observational study'
6	Eligibility criteria	Materials and Methods, paragraph 2: inclusion and exclusion criteria defined
7	On what basis potentially eligible participants were identified	Materials and Methods, paragraph 2: institutional archive records screened for orbital Doppler US performed for suspected brain death
8	Where and when potentially eligible participants were identified (setting, location and dates)	Materials and Methods, paragraph 1: single center (Sakarya University Training and Research Hospital), January 1, 2021 – February 1, 2025
9	Whether participants formed a consecutive, random or convenience series	Materials and Methods, statistical analysis section, final paragraph: 'all eligible patients...were enrolled consecutively'
**METHODS — Test Methods**		

No	STARD 2015 Item	Location in Manuscript
10a	Index test, in sufficient detail to allow replication	Materials and Methods: Samsung HM70 EVO, LA3-16AD transducer (~10 MHz), supine position, closed eyelids, minimal probe pressure, angle correction, standardized protocol; single operator with ~5 years experience
10b	Reference standard, in sufficient detail to allow replication	Materials and Methods, paragraph 3: absence of brainstem reflexes + positive apnea test, documented by responsible clinical physicians
11	Rationale for choosing the reference standard (if alternatives exist)	Materials and Methods, paragraph 3: clinical criteria per institutional protocol; ancillary testing used for additional confirmation where sought
12a	Definition of and rationale for test positivity cut-offs of the index test	Materials and Methods, statistical analysis section: Youden J index used; Results and Figure 1: OA-RI ≥0.77, ONSD ≥4.2 mm
12b	Definition of and rationale for test positivity cut-offs of the reference standard	Materials and Methods, paragraph 3: BD(+) defined by absence of brainstem reflexes and positive apnea test
13a	Whether clinical information and reference standard results were available to the performers/readers of the index test (masking)	Materials and Methods, paragraph 3: classification based on clinical records independently of imaging findings; no masking statement — single-operator retrospective design
13b	Whether clinical information and index test results were available to the assessors of the reference standard (masking)	Materials and Methods, paragraph 3: reference standard established by treating clinical team; imaging results did not influence BD(+)/BD(−) classification
**METHODS — Analysis**		
14	Methods for estimating or comparing measures of diagnostic accuracy	Materials and Methods, statistical analysis section: Mann–Whitney U test, Cliff's delta effect sizes, ROC/AUC (DeLong method), Youden J index for cut-offs
15	How indeterminate index test or reference standard results were handled	Materials and Methods, paragraph 2: cases with technically inadequate images or incomplete

No	STARD 2015 Item	Location in Manuscript
		measurement sets excluded; no imputation applied
16	How missing data on the index test and reference standard were handled	Materials and Methods, paragraph 2: 'only cases with a complete measurement set were included'; no imputation
17	Any analyses of variability in diagnostic accuracy, distinguishing pre-specified from exploratory	Materials and Methods, statistical analysis section: pre-specified hierarchical framework (OA-RI primary, ONSD secondary, others exploratory); FDR correction applied to secondary/exploratory comparisons
18	Intended sample size and how it was determined	Materials and Methods, statistical analysis section, final paragraph: retrospective design; no a priori sample size calculation; consecutive eligible cases; sample size determined by available case pool
**RESULTS — Participants**		
19	Flow of participants, using a diagram	Not included as a formal flow diagram. Patient identification and inclusion/exclusion described in Materials and Methods (paragraphs 1–2) and Results (paragraph 1). A flow diagram may be added if requested by the editor.
20	Baseline characteristics of participants	Results, Table 1 (demographic and clinical characteristics: age, sex, primary diagnosis, apnea test, brainstem reflexes) and Table 2 (ultrasonographic measurements)
21	Distribution of severity of disease in those with the target condition	Table 1: primary clinical diagnoses of BD(+) group (traumatic brain injury 20%, HIE 45%, intracranial hemorrhage 15%, infectious/inflammatory 15%, other 5%)
22	Cross-tabulation of index test results by reference standard results	Results: between-group comparisons in Table 3 (p-values, Cliff's delta, FDR); ROC performance in Table 4
23	Estimates of diagnostic accuracy and their precision (95% confidence intervals)	Results: AUC with 95% CI in text and Table 4; sensitivity and specificity at optimal cut-off; Discussion: cut-off values noted as cohort-specific and exploratory

No	STARD 2015 Item	Location in Manuscript
24	Any adverse events from performing the index test or the reference standard	Not applicable: orbital ultrasonography is non-invasive; no adverse events recorded or reported
**DISCUSSION**		
25	Study limitations, including sources of potential bias, statistical uncertainty, and generalisability	Discussion, limitations paragraph: small BD(−) group, single-center retrospective design, single operator, no inter-observer analysis, single time-point measurements, critically ill comparison group, age-dependent ONSD variability
26	Implications for practice, including the intended use and clinical role of the index test	Discussion (final paragraphs) and Conclusions: OA-RI as bedside adjunct in selected cases; orbital ultrasonography as supportive tool where ancillary testing is indicated
**OTHER INFORMATION**		
27	Registration number and name of registry	Not registered (retrospective observational study; prospective registration not applicable)
28	Where the full study protocol can be accessed	Not applicable (retrospective archive-based study; no prospective protocol)
29	Sources of funding and other support; role of funders	Funding statement: no external funding received; no role of funders

Note: Item 19 (participant flow diagram) may be added as a supplementary figure upon editorial request.
